# Maternal Vitamin D and Inulin Supplementation in Oxidized Oil Diet Improves Growth Performance and Hepatic Innate Immunity in Offspring Mice

**DOI:** 10.3390/antiox12071355

**Published:** 2023-06-28

**Authors:** Guangrong Xie, Qipeng Zhang, Zhengfeng Fang, Lianqiang Che, Yan Lin, Shengyu Xu, Yong Zhuo, Lun Hua, Xuemei Jiang, Jian Li, Mengmeng Sun, Yuanfeng Zou, Chao Huang, Lixia Li, De Wu, Bin Feng

**Affiliations:** 1 Key Laboratory of Animal Disease-Resistant Nutrition of Ministry of Education, Animal Nutrition Institute, Sichuan Agricultural University, Chengdu 611130, China; holl_y@126.com (G.X.); m18483617169@163.com (Q.Z.); zfang@sicau.edu.cn (Z.F.); cheliaqiang@sicau.edu.cn (L.C.); linyan@sicau.edu.cn (Y.L.); shengyuxu@sicau.edu.cn (S.X.); zhuoyong@sicau.edu.cn (Y.Z.); hualun@sicau.edu.cn (L.H.); 71310@sicau.edu.cn (X.J.); 14109@sicau.edu.cn (J.L.); wude@sicau.edu.cn (D.W.); 2 College of Science, Sichuan Agricultural University, Ya’an 625014, China; 14391@sicau.edu.cn; 3 College of Veterinary Medicine, Sichuan Agricultural University, Chengdu 611130, China; yuanfengzou@sicau.edu.cn (Y.Z.); huangchao@sicau.edu.cn (C.H.); 14114@sicau.edu.cn (L.L.)

**Keywords:** oxidative stress, oxidized oil diet, inflammation, toxicology

## Abstract

Dietary oxidized fat contains harmful materials such as hydrogen peroxide and malondialdehyde (MDA). Excessive oxidized fat intake during pregnancy and lactation not only leads to maternal body injury but also damages offspring health. Our previous study demonstrated that vitamin D (VD) had antioxidative capability in sows. This study was conducted to investigate the effect of maternal VD and inulin supplementation in oxidized oil diet on the growth performance and oxidative stress of their offspring. Sixty 5-month-old C57BL/6N female mice were randomly divided into five groups: Control group (basal diet, n = 12), OF group (oxidized-soybean-oil-replaced diet, n = 12), OFV group (oxidized-soybean-oil-replaced diet + 7000 IU/kg VD, n = 12), OFI group (oxidized-soybean-oil-replaced diet + 5% inulin, n = 12) and OFVI group (oxidized-soybean-oil-replaced diet + 7000 IU/kg VD + 5% inulin, n = 12). Mice were fed with the respective diet during pregnancy and lactation. The offspring were then slaughtered on day 21 of age at weaning. Results showed that a maternal oxidized oil diet impaired body weight and liver weight gain of offspring during lactation compared to the control group, while maternal VD, inulin or VD and inulin mixture supplementation reversed this effect. In addition, the activity of T-AOC in the liver of offspring was lower in the OF group than that in the control group, but could be restored by maternal VD and inulin mixture supplementation. Furthermore, the gene expression of both proinflammatory and anti-inflammatory cytokines, such as *Il-6*, *Tnfα* and *Il-10*, in offspring liver were downregulated by a maternal oxidized oil diet compared with the control group, but they were restored by maternal VD or VD and inulin mixture supplementation. The expressions of *Vdr* and *Cyp27a1* were decreased by a maternal oxidized oil diet compared with the control group, while they could be increased by VD or VD and inulin mixture supplementation. Conclusion: maternal oxidized oil diet intake could impair the growth performance by inducing oxidative stress, but this can be relieved by maternal VD and inulin supplementation.

## 1. Introduction

As the popularity of fast-food culture is developing in the 21st century, fried food has taken up a part of people’s lives. Studies have shown that oxidized fats can enter the human chylomicron and become part of the body’s lipid pool, thereby altering the fatty acid composition of body, and finally leading to oxidative stress [1,2]. Mothers experience low oxidative stress themselves during pregnancy [3,4], especially in advanced maternal age [5]. However, an oxidized oil diet not only leads to maternal oxidative stress, but also triggers offspring oxidative stress, characterized by reduced SOD activity in serum [6] and the accumulation of peroxidation products in the liver [7], and those impairments may even persist into adulthood [8]. In addition, maternal oxidative stress during pregnancy can not only lead to maternal organ damage, but also lead to fetal growth arrest, fetal absorption and fetal malformation [9,10]. Therefore, protection of mother and offspring from oxidative stress by enhancing the antioxidative capacity through nutritional methods has become highlighted in recent years.

The main precursors of vitamin D (VD) are ergosterol and 7-hydroxydehydrocholesterol. Under ultraviolet radiation, these two precursors can be converted to VD_2_ and VD_3_, among which VD_3_ is the main form of VD in animals [11]. VD, either obtained from dietary ingredients or from the photochemical and thermal conversion of 7-hydroxydehydrocholesterol in the skin, is transported to the liver in a form that binds to the VD bind protein (DBP), where it undergoes 25-hydroxylation catalyzed by CYP27A1 and other enzymes. It then becomes the active form of 25-OHVD_3_, which is also the common form of VD in the blood. Thus 25-OHVD_3_ is considered to be a marker of the VD content in the body [12]. Besides its primary function in bone growth and calcium homeostasis regulation, VD has been proven to effectively relieve oxidative stress and enhance immunity [13,14]. Some studies have shown that maternal supplementation of VD increased VD level and metabolic-related enzyme activities in their offspring’s organs, such as the brain [15] and liver [16]. In addition, maternal VD addition during the lactation period can improve the intestinal health [17], bone health [18] and immune system development of offspring [19,20]. However, the effect of maternal VD supplementation during pregnancy and lactation on the oxidative status of offspring needs to be explored.

Inulin is a type of natural fructan mixture and is another form of energy storage in plants beside starch. Dietary supplementation of inulin can improve hyperlipidemia and hyperglycemia, and regulate intestinal flora in animals [21]. In recent years, studies on dietary inulin addition in animals during the gestation and lactation periods have shown that maternal inulin addition can decrease oxidative stress level, improve the development of mammary glands and milk ingredients in mothers, and affect the composition of milk to provide better nutrition for lactating offspring [22]. In addition, maternal dietary inulin supplementation can increase a neonate’s tolerance to food antigens and sensitinogens, which supports earlier neonatal adaptation to the environment and diet [22,23]. However, the effect of maternal inulin supplementation during pregnancy and lactation on the oxidative status of offspring needs to be further explored. In addition, whether the benefit of inulin can transport from mother to offspring is not clear.

Dietary supplementation of fructooligosaccharides, such as inulin, is beneficial to the absorption of VD, calcium and phosphorus, which further promotes the bone growth and development of adolescents [24]. In addition, dietary VD or inulin supplementation in gestation has been proven to enhance the gestation outcome [13,14,22,25]. However, the effect of combinatorial VD and inulin supplementation during pregnancy and lactation on the health of offspring is unclear.

This study was conducted to explore whether maternal VD and/or inulin supplementation during pregnancy and lactation could improve the oxidative stress and inflammation in their offspring compared with those raised on a maternal oxidized oil diet.

## 2. Materials and Methods

### 2.1. Experimental Design, Diets, and Management

The animal study protocol was approved by the Animal Care and Use Committee of Sichuan Agricultural University, and the studies were carried out in accordance with the Guide for the Care and Use of Laboratory Animals (National Research Council, Bethesda, MD, USA).

Sixty primiparous C57B6L/N female mice at 5 months of age (Vital River Laboratory Animal Technology Co. Ltd., Beijing, China) were mated with 3-month-old male mice (Vital River Laboratory Animal Technology Co. Ltd., Beijing, China). Female mice were set as day one of pregnancy when a vaginal plug was observed, and were then put in separate cages during the whole gestation and lactation period. Female mice were randomly and averagely assigned into 5 groups on day 1 of gestation: Control group (n = 12), OF group (7% oxidized soybean oil, n = 12), OFV group (7% oxidized soybean oil + 7000 IU/kg VD, n = 12), OFI group (7% oxidized soybean oil + 5% inulin, n = 12) and OFVI group (7% oxidized soybean oil + 7000 IU/kg VD + 5% inulin, n = 12). Female mice were fed with the respective diets, which were based on the recommendation of AIN 93, during the whole gestation and lactation period (Table S1). The inulin was obtained from BENEO-Orafti (Orafti GR, Warcoing, Belgium, purity greater than 90%). The VD was added in the form of VD_3_, which was purchased from Desite (Desite, Chengdu, China, purity greater than 98%). The animal room was set with a 12 h light–dark cycle; temperature was maintained at 22.5 ± 2 °C, and the humidity was maintained at 50–60%.

### 2.2. Preparation of Oxidized Soybean Oil

The soybean oil was barreled food-grade soybean oil (Jinlongyu, Chengdu, China). The oxidized oil for the OF, OFV, OFI and OFVI groups was prepared with soybean oil at 80 °C for 12 h per day using an air pump and continued for 7 days. The peroxide value was then determined according to the national standard of the People’s Republic of China (GB5009. 227-2016). The peroxide concentrations of the fresh soybean oil and the oxidized oil were 2.6 mEqO_2_ kg^−1^ and 245 mEqO_2_ kg^−1^, respectively.

### 2.3. Sample Collection

Birth weights of the pups were measured at delivery. All pups were sacrificed by carbon dioxide anesthesia on day 21 of age at weaning. Body weights and liver weights were measured. Liver samples were quickly frozen in liquid nitrogen and stored at −80 °C for further analysis. Serum samples were harvested by centrifuging the blood and were stored at −20 °C until utilization. The serum and liver samples of 10 pups with body weights around the average BW from each group were selected for enzyme activity and gene expression analysis.

### 2.4. Analysis of Antioxidant and Oxidant Index

Liver samples were rapidly weighed and homogenized in ice cold saline (1:9 of *w*/*v*) using a tissue homogenizer (Bullet Blender, Next Advance, Inc., Averill Park, NY, USA). The homogenate product was then centrifuged at 6000× *g* for 15 min at 4 °C, and the supernatants were used to measure the oxidant and antioxidant index content. Serum samples were centrifuged at 3000× *g* for 15 min at 4 °C. The malondialdehyde (MDA), catalase (CAT), superoxide dismutase (SOD) and glutathione peroxidase (GPH-Px) contents and the total antioxidant capacity (T-AOC) in the supernatants and serum were measured using the respective assay kits (Nanjing Institute of Jiancheng Biological Engineering, Nanjing, China) according to the manufacturer’s instructions.

### 2.5. Assessment of Gene Expression

Total RNA in liver tissue was isolated using TRI reagent (Sigma-Aldrich, Shanghai, China) according to the manufacturer’s instructions. RNA quality was assessed by agarose gel and the concentration was measured with a spectrophotometer (NanoDrop 2000, Thermofisher Scientific, Shanghai, China). A quantity of 1 μg RNA was reverse-transcribed into cDNA with a reverse-transcription PCR kit according to the manufacturer’s instructions (Takara, Dalian, China). Real-time PCR was performed on a quantitative PCR machine (7900HT, ABI, Carlsbad, CA, USA) with Power SYBR Green RT-PCR reagents (BioRad, Hercules, CA, USA). The following reagents were used for each reaction: forward primer, 300 nM; reverse primer, 300 nM; and cDNA sample, 20 ng. The conditions used for PCR were 95 °C for 10 min for 1 cycle, and then 40 cycles of 95 °C for 15 s followed by 60 °C for 1 min. The real-time PCR data were analyzed by the 2-delta delta CT method with β-actin (*Actb*) as the reference gene. The sequences of the primers are listed in Table S2.

### 2.6. Statistical Analysis

All data were expressed as mean ± standard error of the mean (SEM). Body weights, liver weights and average daily body weight gain (ADG) of offspring were analyzed in litters. The OF, OFV, OFI and OFVI groups were compared with the Control group using t-test analysis. A general linear model analysis corrected by the Bonferroni post hoc test was used in the comparison of the oxidized soybean oil groups, including OF, OFV, OFI and OFVI. All these procedures were carried out using IBM SPSS Statistics 22. The value of *p* < 0.05 was considered statistically significant, whereas 0.05 ≤ *p* < 0.1 was considered as a tendency.

## 3. Results

### 3.1. Effects of VD and Inulin Supplementation in an Oxidized Oil Diet during Gestation on Maternal Reproductive Performance

**Figure 1:**
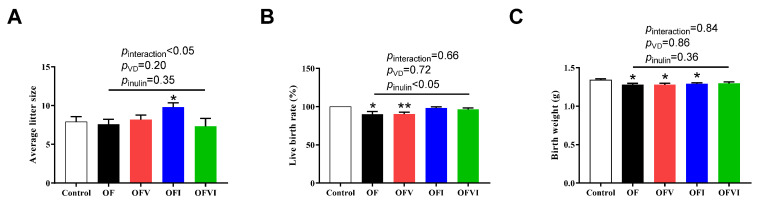
Effects of VD and inulin supplementation in oxidized gestation diet on reproductive performance of female mice. (**A**) Average litter size. (**B**) Live birth rate. (**C**) Birth weight of live-born offspring mice. Data are expressed as mean ± SEM. * *p* < 0.05, ** *p* < 0.01 as compared to Control group.

Though an oxidized oil diet during gestation did not change the body weight and litter size, maternal inulin supplementation in an oxidized oil diet improved litter size compared with the control group. In addition, there was an interaction between VD and inulin addition on the litter size (Figure 1A and Figure S1). The live birth rate and birth weight of the offspring was impaired by maternal oxidized diet regardless of VD supplementation (Figure 1B,C). However, maternal inulin supplementation in an oxidized oil diet during gestation increased the live birth rate (Figure 1B).

### 3.2. Effects of VD and Inulin Supplementation in a Maternal Oxidized Oil Diet on the Growth Performance of Offspring during the Suckling Period

**Figure 2:**
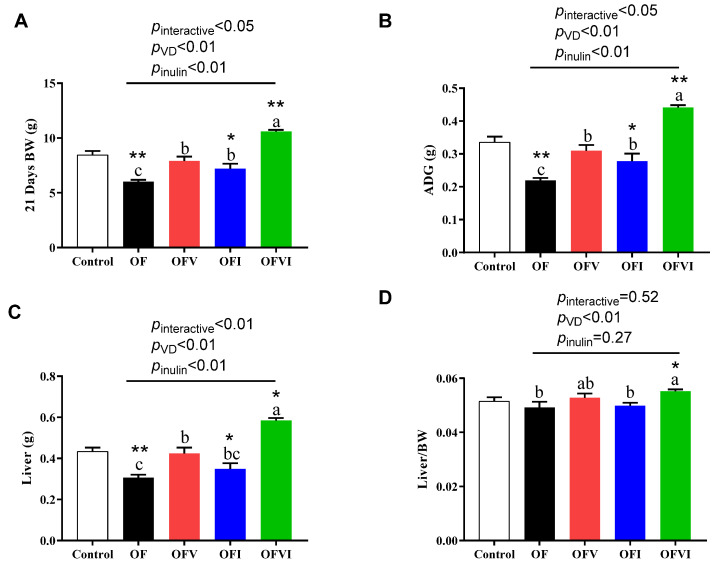
Effects of VD and inulin supplementation in maternal oxidized oil diet on the growth performance of offspring mice during suckling period. (**A**) Body weight of offspring at weaning. (**B**) Average daily body weight gain of offspring during suckling period. (**C**) Liver weight of offspring on d 21 of age at weaning. (**D**) Liver/BW index of offspring at weaning. n = 16–31. Data are expressed as mean ± SEM. * *p* < 0.05, ** *p* < 0.01 as compared to Control group. Different lowercase letters on the bars indicate significant differences among the four oxidized oil diet fed groups (*p* < 0.05).

The maternal oxidized oil diet significantly impaired the weaning body weight (BW), the average daily gain weight (ADG) and weaning liver weight of offspring compared with the control diet, while maternal VD and inulin supplementation restored this effect of the oxidized oil diet (Figure 2A–C). In addition, maternal VD supplementation and inulin supplementation had a synergistic effect on the BW, ADG and liver weight of offspring at weaning (Figure 2A–C). What’s more, maternal VD supplementation increased the liver/BW index of offspring (Figure 2D).

### 3.3. Effects of VD and Inulin Supplementation in a Maternal Oxidized Oil Diet on the Oxidative/Antioxidative Condition of Offspring Mice

**Figure 3:**
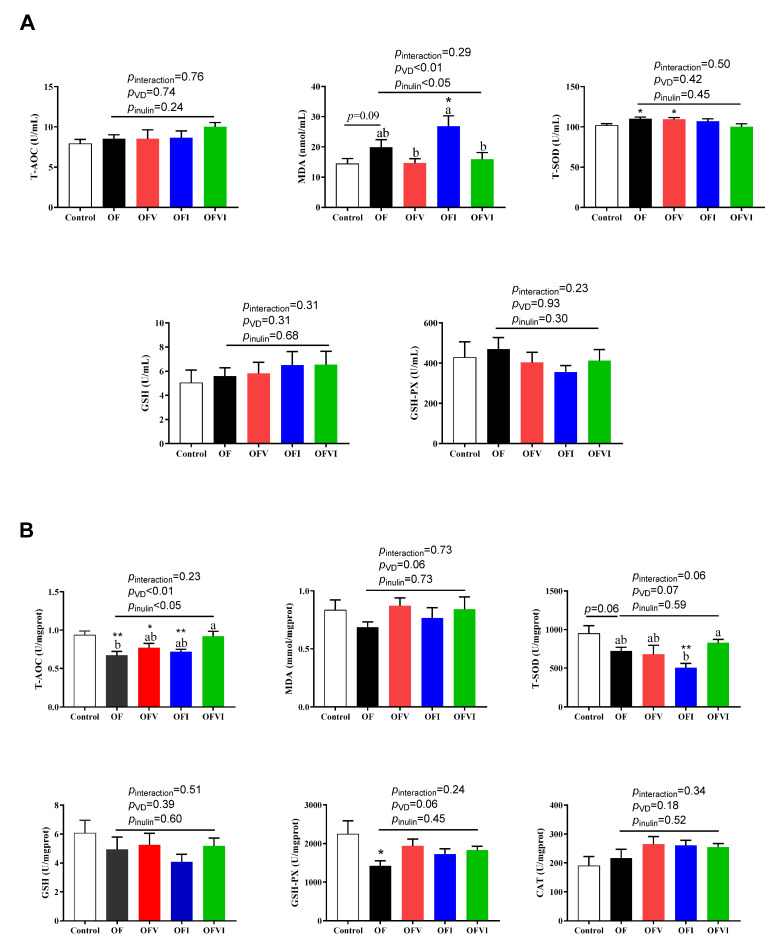
Effects of VD and inulin supplementation in maternal oxidized oil diets on oxidative status of offspring mice. (**A**) The enzyme activities of T-SOD, GSH-Px and levels of T-AOC, MDA and GSH in the serum of offspring. (**B**) The enzyme activities of T-SOD, GSH-Px, CAT and levels of T-AOC, MDA and GSH in the liver of offspring. n = 10 per group. Data are expressed as mean ± SEM. * *p* < 0.05, ** *p* < 0.01 as compared to Control group. Different lowercase letters on the bars indicate significant differences among the four oxidized oil diet fed groups (*p* < 0.05).

The serum level of T-SOD in OF offspring was higher, while MDA tended to be higher, than those in control group offspring (Figure 3A). In addition, maternal VD supplementation decreased the MDA level, while maternal inulin supplementation decreased the T-SOD level, in the serum of offspring (Figure 3A).

In the liver of offspring at weaning, the activities of the antioxidative enzymes T-AOC and GSH-PX were impaired by a maternal oxidized oil diet (Figure 3B). Both maternal VD supplementation and inulin supplementation increased T-AOC activity in the liver of offspring (Figure 3B). In addition, a maternal oxidized oil diet tended to decrease the activity of T-SOD in the liver of offspring compared with the control group (Figure 3B).

### 3.4. Effects of VD and Inulin Supplementation in a Maternal Oxidized Oil Diet on the Expression of Inflammatory Genes in the Liver of Offspring

**Figure 4:**
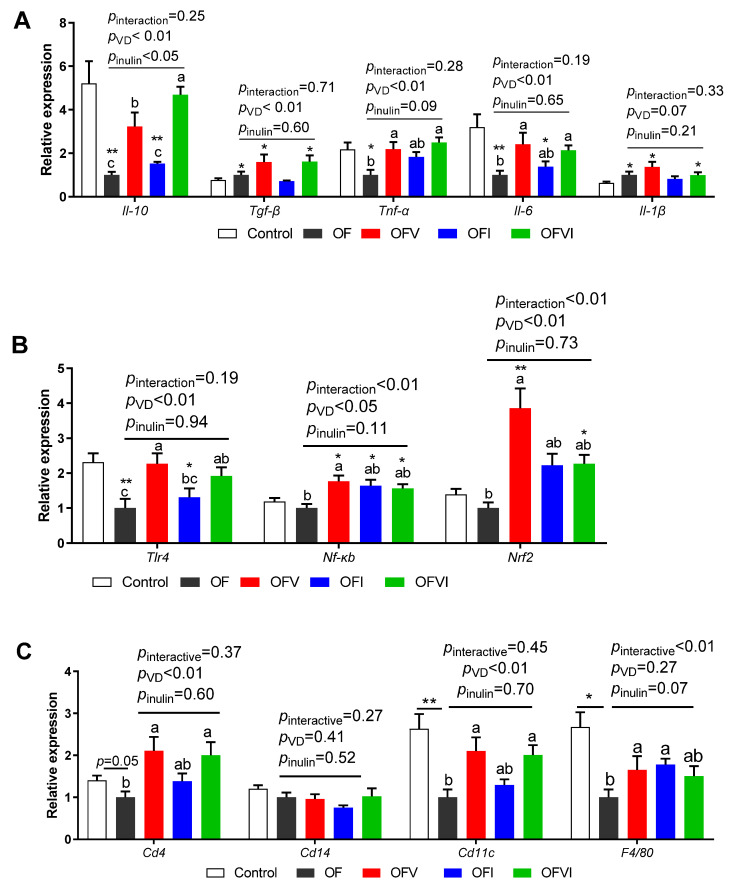
Effects of VD and inulin supplementation in maternal oxidized oil diets on gene expression of inflammatory cytokines, immunity markers and their regulators in offspring liver. (**A**) Gene expression of inflammatory cytokines. (**B**) Gene expression of immunity markers. (**C**) Gene expression of regulators of inflammatory cytokines, immunity markers. Data are expressed as mean ± SEM. * *p* < 0.05, ** *p* < 0.01 as compared to Control group. Different lowercase letters on the bars indicate significant differences among the four oxidized-oil-diet-fed groups (*p* < 0.05).

The mRNA levels of the inflammatory genes *Tnfα* and *Il-6* and anti-inflammatory gene *Il-10* in offspring liver were decreased by a maternal oxidized oil diet as compared with control group, but the decrease could be restored by maternal VD supplementation (Figure 4A). In addition, the expression of *Tgf-β* and *Il-1β* in the liver of offspring was increased by a maternal oxidized oil diet as compared with the control group, regardless of VD supplementation (Figure 4A).

The gene expression of the regulator of immunity and inflammation *Tlr4* in offspring liver was suppressed by a maternal oxidized oil diet as compared with the control group, but this could be reversed by maternal VD supplementation (Figure 4B). In addition, when mothers were fed with an oxidized oil diet, VD supplementation increased the expression of *Nf-kb* and *Nrf2* in the offspring liver, while there was interaction between VD and insulin supplementation on the expression of *Nf-kb* and *Nrf2* (Figure 4B).

For the markers of immune cells, the expression of *Cd4*, *Cd11c* and *F4/80* was suppressed by a maternal oxidized oil diet as compared with the control group, but this could be reversed by maternal VD supplementation (Figure 4C). In addition, maternal inulin supplementation reversed the expression of *F4/80* that was suppressed by a maternal oxidized oil diet (Figure 4C).

### 3.5. Effects of VD and Inulin Supplementation in a Maternal Oxidized Oil Diet on the Expression of VD Metabolism Related Genes in Offspring Liver

**Figure 5:**
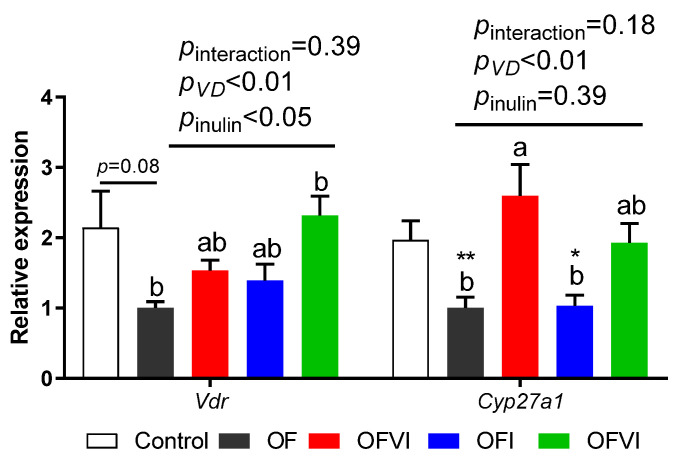
Effects of VD and inulin supplementation in maternal oxidized oil diet on the expression of VD metabolism related gene in offspring liver. Data are expressed as mean ± SEM. * *p* < 0.05, ** *p* < 0.01 as compared to Control group. Different lowercase letters on the bars indicate significant differences among the four oxidized oil diet fed groups (*p* < 0.05).

The gene expression of *Vdr* in offspring liver tended to be suppressed by a maternal oxidized oil diet compared with the control group, and this could be reversed by both maternal VD supplementation and inulin supplementation (Figure 5). In addition, the expression of the main VD metabolism-related gene *Cyp27a* in offspring liver was downregulated by a maternal oxidized oil diet, and this could be reversed by maternal VD supplementation (Figure 5).

## 4. Discussion

Maternal oxidative stress can not only lead to maternal organ damage, but also have long-term effects on offspring [26,27,28]. Similarly to previous studies, a maternal oxidized oil diet not only decreased the birth weight and increased the stillbirth rate, but also decreased the body weight and liver weight in offspring at weaning. Furthermore, we also found that the lower weight of pups caused by a maternal oxidized oil diet could be improved by maternal VD and inulin. In addition, maternal VD supplementation and inulin supplementation had a synergistic effect on the BW, ADG and liver weight of offspring at weaning. Weaning weight is an important factor affecting later growth and health. Holanda et al. found that nursery pigs with light weaning body weight were more susceptible to jejunal inflammation and had impaired intestinal health due to weaning stress [29]. In addition to antioxidant effects, the supplementation of inulin and VD can improve the development of animals’ mammary glands and the composition of milk, which can provide better nutrition for lactating offspring [22,30]. Insufficient vitamin D intake during pregnancy has been found to reduce fetal birth weight [31]. The addition of fructooligosaccharides, such as inulin, is beneficial to the absorption of vitamin D, calcium and phosphorus, which further promotes the bone growth and development of adolescents [24]. In this study, we found that there is a synergistic effect of VD and inulin supplementation on the weaning body weight and liver weight of offspring at weaning.

We previously reported that VD supplementation during gestation improved the litter size of sows [17]. However, it was shown that maternal VD supplementation in an oxidized oil diet did not improve the litter size, birth weight or live birth rate of offspring. The difference might be because of the difference in species and time of treatment. For the sow study, the treatment time lasted for 110 days, while it was 21 days for mouse study. Higher doses or longer-term treatments of VD will be studied on mice in the future. In addition, maternal inulin supplementation during gestation has an increase effect on the live birth rate of offspring; this is similar to the previous report no difference in litter size from ICR mice with 0, 0.25, 1 or 4 µg/kg VD_3_ in drinking water (where 1 IU is defined as the biological activity of 0.025 µg cholecalciferol) [32].

Maternal oxidative stress can change the milk nutrition composition [33], which might cause offspring oxidative stress and impair organ health before weaning [34,35,36]. In this study, we found that a maternal oxidized oil diet increased serum MDA content, while decreasing hepatic T-AOC, SOD and GSH-px activities in offspring. Dysregulation of antioxidant homeostasis is associated with the occurrence of many diseases, while increased levels of antioxidant enzymes can effectively repair DNA damage, and may reduce or alleviate the onset of chronic diseases [37], such as chronic obstructive pulmonary disease [38], atherosclerosis of the arteries [39] and chronic kidney disease [40]. Numerous studies have proven that dietary inulin [41,42] and VD [43,44] addition can improve immunity and anise oxidative stress through activation of Nrf2 and its downstream antioxidant enzyme system, including GSH-px, SOD, CAT, etc. In addition to alleviating oxidative stress, the activation of Nrf2 can also regulate the inflammatory state and reduce the occurrence of ferroptosis, which plays an important role in inflammation [45], cancer [46], aging [47], cell damage [48], fertility [49] and other aspects. We found the antioxidant function to be more effective with both VD and inulin mixture supplementation in diet, as the results in our study showed that offspring from the OFVI group had higher hepatic T-AOC and SOD activities than those in the other groups. In addition, we observed that the expression of antioxidant factor Nrf2 in offspring liver was suppressed by a maternal oxidative oil diet, but could be restored by maternal VD supplementation. The specific mechanism of the antioxidative function of VD and inulin and their interaction need to be further studied.

Continuous oxidative stress can induce lipid peroxidation by producing a large amount of ROS, which in turn causes mitochondrial damage and cell apoptosis and reduces the body’s inflammatory response [50,51]. Wang et al. [52] reported that maternal oxidized soybean oil addition during gestation and lactation decreased both pro-inflammatory and anti-inflammatory genes in offsprings’ jejunums on d 21 of age. We also observed that a maternal oxidized oil diet decreased the gene expression level of inflammatory cytokines *Il-6* and *Tnf-α* in the livers of offspring at weaning. In addition, the expression of anti-inflammatory gene *Il-10* in offspring liver was also decreased by a maternal oxidized oil diet. It has been reported that pro-inflammatory cytokines, such as IL-6 and TNF-α, can drive innate immunity [53]. In addition, we also observed that maternal dietary oxidized oil intake decreased the expression of *Tlr4*, one of the typical pattern receptors. TLR4 is one of the most intensively studied TLRS, which can recognize the surface ligands of Gram-negative bacteria and initiate the innate immune response [54]. In the liver, TLR4 is expressed on the surface of hepatocytes [55], Kupffer cells (KCs) [56], hepatic dendritic cells (HDCs) [57] and other cells [58]. Activation of TLR4 leads to an inflammatory cascade that induces the expression of inflammatory cytokines, such as IL-6, IL23 and TNF-α, through its downstream gene NFκB, and finally leads to pathogen clearance [59]. Moreover, the TLR4-NF-κB pathway also regulates the maturation of immune cells [60]. Because of the rich in variety of immune cells, the liver is also one of the main organs for inflammatory response [61]. The abnormal expression of TLR4 and NF-κB causes some liver disease [56]. We observed that the expression of some immune cell markers in offspring liver, such as Cd4, F4/80 and Cd11c, were downregulated by a maternal oxidized oil diet compared with the control group. Hallworth et al. [62,63] reported that insufficient phagocytic capacity, insufficient expression of TLR4 and low levels of cytokine production of monocytes are closely related to the susceptibility to infection and development of sepsis in very low birth weight (VLBW) infants. Thus, our data indicates that maternal oxidative oil diet might impair the innate immunity of offspring. However, this needs further study to be confirmed.

Dietary inulin addition is observed to induce liver reparation [64]. In addition, dietary inulin supplementation during gestation has been proven to be beneficial for infant development [65]. Inulin is known for its function in regulating intestinal immunity. In addition, there is close interaction between the gut and liver, which is called the gut-liver axis [66]. Thus, inulin also plays a role in improving liver immunity and alleviating many liver diseases such as alcoholic liver disease [67] and non-alcoholic fatty liver disease [68]. Furthermore, maternal VD supplementation can improve offspring response to inflammatory stimulants such as LPS and CpG [20]. Furthermore, the addition of inulin was also found to regulate calcium homeostasis and alleviate liver injury [69]. In our study, maternal VD and inulin supplementation during gestation and lactation restored the expression of pro-inflammatory and anti-inflammatory genes and their regulators, which were downregulated by a maternal dietary oxidized oil diet. This may relate to some congenital immune response, but the specific mechanism of this change needs further study.

We also found that maternal oxidized oil intake decreased *Vdr* and 25-α vitamin D hydroxylase *Cyp27a1* gene expression in the liver of offspring. In addition, the expression of inflammatory genes in the livers of offspring was also increased by a maternal oxidized oil diet, similarly to previous reports [70,71]. The expression of *Vdr* not only related to VD insufficiency, but also related to the occurrence some diseases, such as cardiovascular disease (CD) [72], oral cancer [73], etc. Our study showed that the supplementation of inulin and VD in the maternal diet could upregulate the gene expression of *Vdr* and *Cyp27a1*. However, whether the increase of *Vdr* and *Cyp27a1* expression is the key pathway of maternal VD and inulin improving the growth and health of offspring or not needs to be further clarified.

To sum up, we found that maternal inulin and VD dietary supplementation under an oxidized oil diet can improve the weaning body weight and liver weight of offspring, and alleviate oxidative stress and inflammation, and enhance hepatic-immunity-related gene expression, in offspring. This study provides a theoretical basis for alleviating offspring disorders derived from maternal oxidative stress by improving maternal VD and inulin nutrition. Future study may focus on understanding the mechanism that mediates the improvement of offspring growth and immunity by maternal VD and inulin supplementation.

## 5. Conclusions

A maternal oxidized oil diet during gestation and lactation may impair the growth performance and hepatic innate immunity of their offspring, but this could be improved by maternal VD and inulin supplementation.

## Data Availability

The data presented in this study are available on request from the corresponding author.

## Supplementary Materials

The following supporting information can be downloaded at: https://www.mdpi.com/article/10.3390/antiox12071355/s1, Figure S1: Maternal body weight during gestation; Table S1: Composition of the diet; Table S2: Primer sequences of the target and reference genes.
