# Supplementary material for: Maternal Vitamin D and Inulin Supplementation in Oxidized Oil Diet Improves Growth Performance and Hepatic Innate Immunity in Offspring Mice
Source: Antioxidants (Basel). 2023 Jun 28;12(7):1355. doi: 10.3390/antiox12071355 (PMC10376903; doi:10.3390/antiox12071355)

Supplementary

Table S1. Composition of the diet

Ingredient	Control(%)	OF(%)	OFV(%)	OFI(%)	OFVI(%)
Casein	20	20	20	20	20
Maltodextrin	10	10	10	10	10
Cornstarch	46.01	46.01	46.01	46.01	46.01
Microcrystalline cellulose	5	5	5		
Inulin	-	-	-	5	5
Soy bean oil	7	-	-	-	-
Oxidized Soybean oil	-	7	7	7	7
Sucrose	8	8	8	8	8
Choline chloride	0.26	0.26	0.26	0.26	0.26
L-Cystine	0.3	0.3	0.3	0.3	0.3
Calcium hydro phosphate	1	1	1	1	1
Potassium citrate	0.8	0.8	0.8	0.8	0.8
Calcium carbonate	0.6	0.6	0.6	0.6	0.6
Mineralelement[1]	1	1	1	1	1
Vitamincomplex[2]	0.03	0.03	0.03	0.03	0.03
Total	100	100	100	100	100
composition（%）					
Carbohydrate	64.01	64.01	64.01	64.01	64.01
Crude fat	7	7	7	7	7
Crude protein	17	17	17	17	17

[1] Mineral elements per kg diet: calcium 5000 mg; Phosphorus 1561 mg; Potassium 3600 mg; Sodium 1000 mg; Chlorine 1600 mg; Magnesium 500 mg; Sulfur 330 mg; Iron 37 mg; Zinc 29 mg; Manganese 59 mg; Copper 6 mg; Iodine 0.2 mg, and the rest was filled with the carrier.

[2] Vitamin content per kilogram of diet: VA 4000 IU; VD3 1000 IU; VE 75 IU; VK 30.75 mg; VB 15.0 mg; VB 26.0 mg; VB 66.0 mg; VB 120.025 mg; Niacin 30.0 mg; D-calcium pantothenate 15.3 mg; Folic acid 2.0 mg; Biotin 0.2 mg.

Table S2. Primer sequences of the target and reference genes

Primer	Accession.no		Sequence(3’→5’)
β-actin	NM_007393.4	F	GGCTGTATTCCCCTCCATCG
		R	CCAGTTGGTAACAATGCCATGT
Nrf2	AH006764	F	TCTTCCATTTACGGAGACCCAC
		R	ACATTGGGATTCACGCATAGGA
Cyp27a1	NM_024264.5	F	AAGGGCCTCACCTATGGGAT
		R	CACCTGGTCCCCTGATTCAC
Vdr	NM_009504.4	F	TCCCCCATCCCTAGAACCAG
		R	TCCTCATTGCTTGGGCTCTG
Tlr4	NM_021297.3	F	TGCCACCAGTTACAGATCGTC
		R	GGGACTTTGCTGAGTTTCTGA
Nf-κb	NM_199267.2	F	ATGGCAGACGATGATCCCTAC
		R	ATGGCAGACGATGATCCCTAC
Cd14	NM_009841.4	F	CTCTGTCCTTAAAGCGGCTTAC
		R	GTTGCGGAGGTTCAAGATGTT
Il-6	NM_012589.2	F	TAGTCCTTCCTACCCCAATTTCC
		R	TTGGTCCTTAGCCACTCCTTC
Il-10	NM_010548.2	F	AGCCTTATCGGAAATGATCCAGT
		R	GGCCTTGTAGACACCTTGGT
Il-1 β	NM_008361	F	GAAATGCCACCTTTTGACAGTG
		R	TGGATGCTCTCATCAGGACAG
Tnf-α	NM_013693.3	F	CATCTTCTCAAAATTCGAGTGACAA
		R	TGGGAGTAGACAAGGTACAACCC
Cd11c	NM_021334.2	F	GCACACTGTGTCCGAACTCA
		R	CTGGATAGCCTTTCTTCTGCTG
F4/80	NM_010130.4	F	CCTCTTCTGGGGCTTCAGTG
		R	TGCAGACTGAGTTAGGACCAC
Cd4	NM_013488.3	F	TTTCTCTGCCTAGCCCCTGA
		R	GAGTAGTCGTGAGGGTCCCA

Fig S1 Maternal body weight during gestation

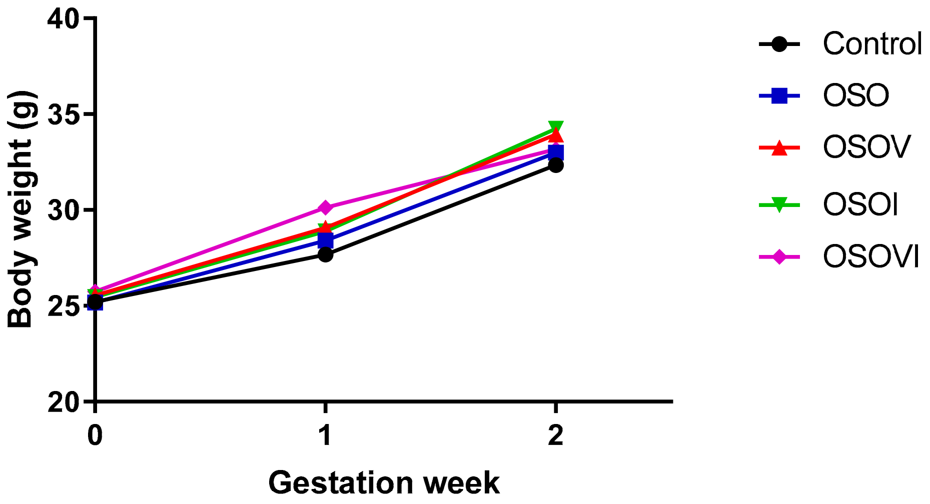

Supplement: Supplementary file 1 [file antioxidants-12-01355-s001.zip › antioxidants-2457198-supplementary.pdf]
